# Left Ventricular Function and Myocardial Triglyceride Content on 3T Cardiac MR Predict Major Cardiovascular Adverse Events and Readmission in Patients Hospitalized with Acute Heart Failure

**DOI:** 10.3390/jcm9010169

**Published:** 2020-01-08

**Authors:** Kuang-Fu Chang, Gigin Lin, Pei-Ching Huang, Yu-Hsiang Juan, Chao-Hung Wang, Shang-Yueh Tsai, Yu-Ching Lin, Ming-Ting Wu, Pen-An Liao, Lan-Yan Yang, Min-Hui Liu, Yu-Chun Lin, Jiun-Jie Wang, Koon-Kwan Ng, Shu-Hang Ng

**Affiliations:** 1 Department of Radiology, Chang Gung Memorial Hospital, Keelung and Chang Gung University, Keelung 20401, Taiwan; kc1116@cgmh.org.tw (K.-F.C.); yuching1221@cgmh.org.tw (Y.-C.L.); 2 Department of Medical Imaging and Intervention, Chang Gung Memorial Hospital, Linkou and Chang Gung University, Taoyuan 33305, Taiwan; giginlin@cgmh.org.tw (G.L.); spookie@cgmh.org.tw (P.-C.H.); 8801131@cgmh.org.tw (Y.-H.J.); wakefield1006@gmail.com (P.-A.L.); jack805@gmail.com (Y.-C.L.); jiunjie.wang@gmail.com (J.-J.W.); 3 Imaging Core Lab, Institute for Radiological Research, Chang Gung University, Taoyuan 333, Taiwan; 4 Clinical Metabolomics Core Lab, Chang Gung Memorial Hospital, Taoyuan 333, Taiwan; 5 Department of Cardiology and Heart Failure Center, Chang Gung Memorial Hospital, Keelung 20401, Taiwan; bearty54@gmail.com (C.-H.W.); khfoffice@gmail.com (M.-H.L.); 6 Graduate Institute of Applied Physics, National Chengchi University, Taipei 11605, Taiwan; syytsai@gmail.com; 7 Department of Radiology, Kaohsiung Veterans General Hospital, Kaohsiung 81362, Taiwan; wu.mingting@gmail.com; 8 Clinical Trial Center, Chang Gung Memorial Hospital at Linkou, Taoyuan 333, Taiwan; lyyang0111@gmail.com

**Keywords:** cardiac magnetic resonance imaging, heart failure, left ventricular systolic function, magnetic resonance spectroscopy, myocardial triglyceride content

## Abstract

Background: This prospective study was designed to investigate whether myocardial triglyceride (TG) content from proton magnetic resonance spectroscopy (MRS) and left ventricular (LV) function parameters from cardiovascular magnetic resonance imaging (CMR) can serve as imaging biomarkers in predicting future major cardiovascular adverse events (MACE) and readmission in patients who had been hospitalized for acute heart failure (HF). Methods: Patients who were discharged after hospitalization for acute HF were prospectively enrolled. On a 3.0 T MR scanner, myocardial TG contents were measured using MRS, and LV parameters (function and mass) were evaluated using cine. The occurrence of MACE and the HF-related readmission served as the endpoints. Independent predictors were identified using univariate and multivariable Cox proportional hazard regression analyses. Results: A total of 133 patients (mean age, 52.4 years) were enrolled. The mean duration of follow-up in surviving patients was 775 days. Baseline LV functional parameters—including ejection fraction, LV end-diastolic volume, LV end-diastolic volume index (LVEDVI), and LV end-systolic volume (*p* < 0.0001 for all), and myocardial mass (*p* = 0.010)—were significantly associated with MACE. Multivariable analysis revealed that LVEDVI was the independent predictor for MACE, while myocardial mass was the independent predictor for 3- and 12-month readmission. Myocardial TG content (lipid resonances δ 1.6 ppm) was significantly associated with readmission in patients with ischemic heart disease. Conclusions: LVEDVI and myocardial mass are potential imaging biomarkers that independently predict MACE and readmission, respectively, in patients discharged after hospitalization for acute HF. Myocardial TG predicts readmission in patients with a history of ischemic heart disease.

## 1. Introduction

Heart failure (HF) is a complex clinical syndrome that results from a variety of conditions preventing the left ventricle (LV) from supporting physiological circulation [1]. Patients with HF are at an increased risk of major adverse cardiovascular events (MACE)—including death, myocardial infarction, stroke, and hospitalizations [2,3]—which pose a significant public health burden globally. The 3-month readmission rates of patients with acute HF remains as high as 25–50%, with 5-year survival rates <50% [4]. Optimized risk sFCtratification would help to prioritize the surveillance for those who are prone to experience MACE.

Cardiomyocytes primarily depend on the oxidation of fatty acids as their source of energy [5]. Because the heart does not serve as a storage depot for fat, the concentration of triglycerides (TG) in the myocardium is low under physiological conditions. However, cardiac steatosis may develop as a result of abnormal regulation of fatty acids uptake or alterations in lipid metabolism. TG accumulation in the heart has been recognized as a risk factor for cardiovascular (CV) disease [6,7,8]. Vilahur et al. have shown that intramyocardial lipids impaired myofibroblast-related collagen synthesis with resultant poor healing of the myocardial scar post-myocardial infarction, while excess cardiac lipids exacerbated apoptosis and led to extensive myocardial infarcts [9]. Proton magnetic resonance spectroscopy (^1^H-MRS) has been used to obtain in vivo quantitative measures of myocardial TG content in patients with CV disorders [10,11,12]. We have previously used 3.0 T CMR to analyze the association between myocardial unsaturated fatty acids (UFA) content and left ventricular (LV) function in patients who had been hospitalized for acute HF [13]. Furthermore, cardiac magnetic resonance imaging (CMR) is well established as the gold standard in the assessment of cardiac structure and function, with incremental diagnostic and prognostic information in HF [14,15,16]. We, therefore, hypothesize that the quantitative information about myocardial TG content from ^1^H-MRS and LV function parameters from CMR may help predict the occurrence of future MACE and readmission in patients who have been hospitalized for acute HF.

This prospective study was designed to investigate whether myocardial TG content from ^1^H-MRS (primary aim) and LV function parameters from CMR (secondary aim) can serve as imaging biomarkers in predicting future MACE and readmission in patients who have been hospitalized for acute HF.

## 2. Methods

### 2.1. Ethics Approval and Consent to Participate

Ethical approval was granted by the Institutional Review Board of the Keelung Chang Gung Memorial Hospital (IRB 102-2772A3). The study is reported in accordance with the STROBE (STrengthening the Reporting of OBservational studies in Epidemiology) statement and has been registered at ClinicalTrials.gov (Identifier: NCT02378402) on 21 February 2015. All patients provided their written informed consent.

### 2.2. Study Design and Patient Population

**Figure 1:**
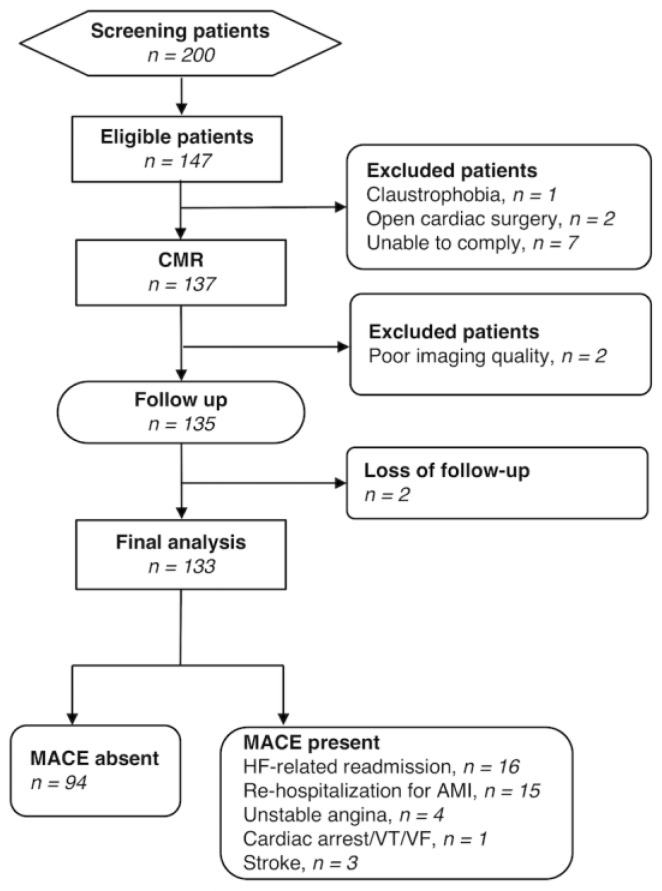
Flow diagram of the study cohort. Note.—AMI, acute myocardial infarction; CMR, cardiac magnetic resonance; HF, heart failure; MACE, major cardiovascular event; VT, ventricular tachycardia; VF, ventricular fibrillation.

Between March 2014 and June 2016, we prospectively screened 200 patients and enrolled a total of 147 patients who were hospitalized with acute HF at a tertiary referral hospital with a dedicated HF center (IRB 102-2772A3, ClinicalTrial.gov: NCT02378402). Patients were scanned whose medical condition had become stable after treatment, specifically when they (1) had an oral medication regimen stable for at least 24 h; (2) had no intravenous vasodilator or inotropic agents for at least 24 h; and (3) were ambulatory before discharge to assess functional capacity. Patients aged between 20 and 70 years with acute HF, with initial HF stage C, classified according to the American College of Cardiology (ACC) and the American Heart Association (AHA) HF classification system [4], were eligible. Patients who were unwilling to participate or presenting with general contraindications to CMR (e.g., claustrophobia, metal-containing implants, cardiac pacemakers, or unable to comply with the examiners) were excluded. Additional exclusion criteria were as follows: Positive history of open cardiac surgery; pregnancy or breastfeeding; and inability to adhere to treatment and/or follow-up. All of the medical records underwent a central review by a multidisciplinary team to confirm that the identified patients were suitable for inclusion. The following variables were collected at baseline: Demographic data (age, sex, height, and weight), cardiovascular risk factors (smoking, hypertension, diabetes), previous history of cardiovascular disease (angina, myocardial infarction, dilated cardiomyopathy, myocarditis), and medication use. Serum lipid levels (total cholesterol, very-low-density lipoprotein cholesterol, low-density lipoprotein cholesterol, high-density lipoprotein cholesterol, and TG) were measured 1 month before CMR imaging. Patients with a previous history of angina or myocardial infarction were defined as an ischemic group, while the others as non-ischemic group. Previously, 48 of the 133 patients have been reported in a cross-sectional interim report to study the association of LV function and myocardial TG on CMR [13]. In this study, we further evaluated their predictive value in a longitudinal observational study. The patient flow diagram is present in Figure 1.

### 2.3. H-MRS

Clinical MRS was acquired before contrast administration using the same settings and anatomical localizations as previously reported [13]. In brief, respiratory-triggered cardiac-gated point-resolved spectroscopy (PRESS) [17] was implemented to acquire localized ^1^H MRS voxels to a 2 × 2 × 1 cm^3^ spectroscopic volume within the interventricular septum during the end-systolic phase. The MRS acquisition parameters were as follows: Nominal TR (repetition time)/TE (echo time), 550 ms/33 ms; 64 averages; window size, 1024 points; bandwidth, 2000 Hz [18,19,20,21]. Spectra with and without water suppression were used to obtain water and myocardial TG signals, respectively [22].

### 2.4. CMR Imaging

Patients were required to fast overnight before undergoing CMR examinations on a 3.0-Tesla Siemens Skyra MR scanner (Siemens, Erlangen, Germany). No pre-medications were used. The scanner was equipped with an 18-channel phased-array receiver body coil and operated on a VD13 platform. Steady-state free precession (SSFP) cine imaging was used to produce images with both short-axis (contiguous 8 mm slice thickness) and standard long-axis views (2-, 3- and 4-chamber views). Late gadolinium enhancement (LGE) at 10 min after gadolinium injection in the short-axis (9 to 13 images covering the entire LV), 2-chamber, and 4-chamber planes. The following settings were employed: Echo time, 1.2 ms; repetition time, 3.4 ms; field of view, 34−40 cm; matrix, 256 × 256.

### 2.5. Image Analysis

Left ventricular ejection fraction (LVEF) was measured on short-axis cine LV images with post-processing software (VB17, Argus Viewer and Function, Siemens, Erlangen, Germany) on a separate workstation. LV endocardial and epicardial borders were manually drawn at end-diastole and end-systole on short-axis cine images and LVEF and end-diastolic LV mass from each slice were measured accordingly. Papillary muscles were not included in the LV mass. Left ventricular end-diastolic volume (LVEDV) and end-systolic volume (LVESV) were calculated using the same methodology. The left ventricular end-diastolic volume index (LVEDVI) was determined by dividing the LVEDV by the body surface area (BSA). The LV global function index (LVGFI) was calculated with the following formula:

LVGFI = [LVESV/(LVEDV+LVESV)/2 + (LV mass/1.05)] × 100(1)

**Figure 2:**
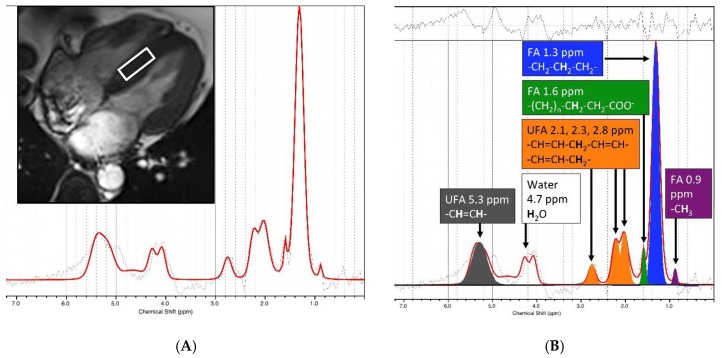
Example myocardial CMR spectroscopy. (**A**) A 2 × 2 × 1 cm^3^ spectroscopic volume (white box) was acquired from the interventricular septum during the systolic phase to generate an input spectrum. (**B**) ^1^H-CMR spectra were fitted and analyzed using the LCModel 6.2 software package (right). We quantified the components of myocardial triglyceride resonances, i.e., fatty acids (FA, lipid resonances δ 0.9, 1.3, and 1.6 ppm) and unsaturated fatty acids (UFA, lipid resonance δ 2.1 and 2.3, 2.8, 5.3 ppm).

LCModel 6.2 software package (http://s-provencher.com/pages/lcmodel.shtml) was used for the quantification of TG by fitting the time-domain ^1^H-MRS spectra (Figure 2). Multiple resonance peaks of fat including methyl (–(CH_2_)_n_–CH_3_) peak δ 0.9 ppm, methylene (–(CH_2_)_n_–) peaked at 1.3 ppm, beta-carboxyl (–CO-CH_2_-CH_2_–) at 1.6 ppm, alpha-allylic (–CH_2_–CH=CH–CH_2_–) peaked at 2.02 ppm (denote by Lip2.1), alpha-carboxyl (–CO-CH_2_-CH_2_–) peaked at 2.24 ppm (denote by Lip2.3), diacyl (–CH=CH–CH_2_–CH=CH–) peaked at 2.75 ppm (denote by Lip2.8), olefinic (–CH=CH–) at 5.29 ppm (denote by Lip5.3) were fitted. Water-suppressed spectra were used to quantify the total myocardial TG resonance and its—including FA (lipid resonances δ 0.9, 1.3 and 1.6 ppm) and UFA (lipid resonance δ 2.02 ppm, 2.24 ppm, 2.75 ppm, and 5.29 ppm), i.e., the ratio of the metabolite resonance area to the unsuppressed water resonance area. Water resonance (~δ 4.7 ppm) without water suppression was also determined for normalization. Cramer-Rao lower bound (CRLB) of TG provided by the LCModel was served as a goodness-of-fit, and used for the evaluation of the spectra quality.

### 2.6. Treatment and Definition of the Study Outcomes

Patients were clinically followed on a monthly basis by a dedicated HF team, who were aware of conventional CMR but blinded to the MRS results. MACE is a composite of clinical events without standard definition, because individual outcomes used to make this composite endpoint vary by study [2,3,23]. According to our study endpoints, we used the term MACE to comprise the composite of the events including HF worsening, HF-related readmissions, cardiac catheterization, unstable angina, stroke, cardiac arrest/ventricular tachycardia (VT)/ventricular fibrillation (VF), and cardiac death. However, the HF related-readmission rates were not included in MACE and, thus, were separately considered. The appropriate length of the 2-year follow-up was determined based on our previous heart failure cohort study [24].

### 2.7. Data Analysis

Univariate and stepwise multivariable regression analyses (Wald statistics) were used to assess CMR parameters. A complete-case analysis was implemented (missing data were not excluded). Survival curves were plotted with the Kaplan-Meier method (log-rank test). Two-group comparisons of continuous and categorical variables were performed with the Student’s *t*-test (two-group comparisons) and chi-square test, respectively. Continuous variables were determined by the recursive partitioning method to obtain the optimal cut-off values. Independent predictors of MACE and readmission rates were identified using univariate and multivariable Cox proportional hazard regression analyses. A Bonferroni posthoc correction was conducted to reduce Type I Error by dividing the original α-value by the number of analyses on the dependent variable. Data correlation was evaluated based on the Spearman rank test. Data analyses were performed using the following software: SPSS (version 11; SPSS Inc., Chicago, IL, USA), MedCalc (version 9.2.0.0; MedCalc Software, Mariakerke, Belgium), and R (version 3.5.3, R Foundation for Statistical Computing, Vienna, Austria, www.r-project.org).

## 3. Results

### 3.1. Patient Characteristics

A total of 133 consecutive patients (mean age, 52.4 years) entered the final analysis, with the mean follow-up time for surviving patients being 775 days. Table S1 details the baseline LV functional parameters. The baseline LVEF of this patient population was 52.2 (52.2 ± 21.7%). There were more patients with preserved EF ≥55% (*n* = 71) than reduced EF <55% (*n* = 62). MACE was observed in 39 cases (29.3%). The MACE with their distribution being as follows: HF-related readmission (*n* = 16; ischemic/non-ischemic 6/10), re-hospitalization for acute myocardial infarction (*n* = 15; 15/0), unstable angina (*n* = 4; 4/0), cardiac arrest (*n* = 1; 1/0), and stroke (*n* = 3; 0/3). There were no cases of cardiac death. The baseline clinical characteristics of the study participants are summarized in Table 1. Patients who experienced MACE did not differ from those who did not in terms of baseline clinical characteristics. As far as HF-related events are concerned, 6 patients (4.5%) were readmitted within 3 months and 18 patients (13.5%) within 12 months. Only one patient (0.8%) was readmitted within 30 days. Ischemic heart disease was identified in 50 patients, with the involvement of the left main (*n* = 2), left anterior descending (*n* = 36), left circumflex (*n* = 26), and right coronary artery (*n* = 23), verified by the presence of a myocardial scar on LGE. The remaining 83 patients had no history of ischemic heart disease and had no myocardial scar on LGE.

### 3.2. Associations between CMR, and ^1^H-MRS Parameters with MACE and HF-Related Readmission

**Figure 3:**
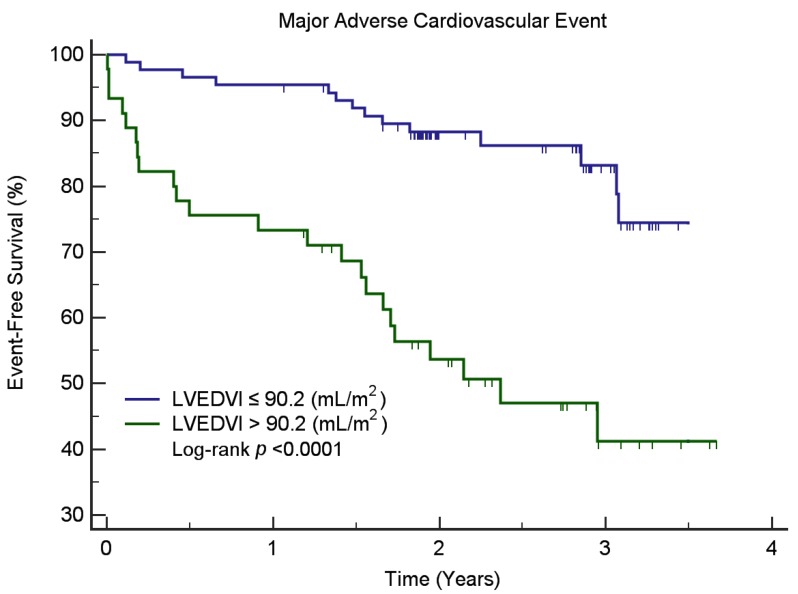
Kaplan-Meier curves for MACE-free survival in patients stratified according to the left ventricular end-diastolic volume index (LVEDVI) on CMR. Note—Kaplan-Meier survival analysis demonstrated that all patients with low LVEDVI (≤90.2 mL/m^2^) had a lower probability for MACE than those with high LVEDVI (>90.2 mL/m^2^, log-rank test, *p* < 0.0001). MACE, major cardiovascular event.

All of the CMR parameters (EF, LVEDV, LVEDVI, LVESV, LV mean cavity volume, LV global volume, and LVGFI) were reciprocally correlated with one another (*p* < 0.0001). The results of univariate Cox regression analysis for MACE and HF-related readmission are shown in Table 2 and Table 3. After allowance for potential confounders in multivariable analysis, LVEDVI was identified as an independent predictor for MACE, whereas myocardial mass independently predicted 3- and 12-month readmission rates. Kaplan-Meier survival analysis (Figure 3) demonstrated that patients with low LVEDVI (≤90.2 mL/m^2^) had a lower probability for MACE than those with high LVEDVI (>90.2 mL/m^2^, log-rank test, *p* < 0.0001).

**Figure 4:**
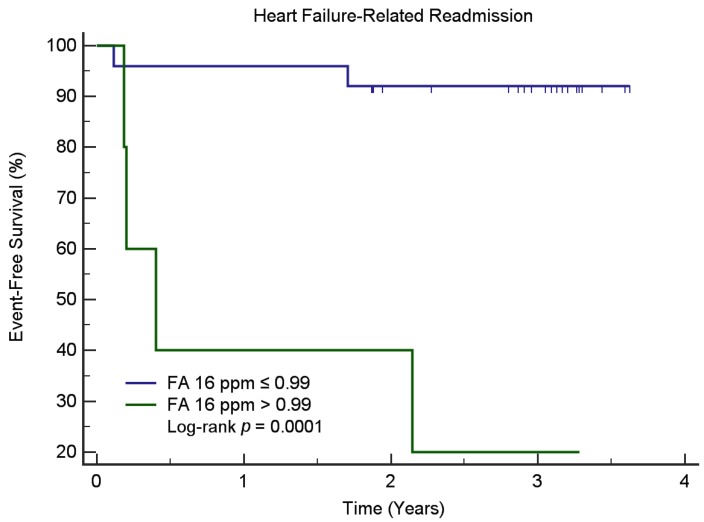
Kaplan-Meier curves for readmission-free survival in ischemic patients stratified according to the level of lipid resonances δ 1.6 ppm on ^1^H-MRS. Note—Kaplan-Meier survival analysis demonstrated that ischemic patients with low levels of lipid resonances δ 1.6 ppm (≤0.99) had a lower probability for heart failure-related readmission than those with high levels of lipid resonances δ 1.6 ppm (>0.99, log-rank test, *p* < 0.0001). Note.—Abbreviations: FA, fatty acid.

**Figure 5:**
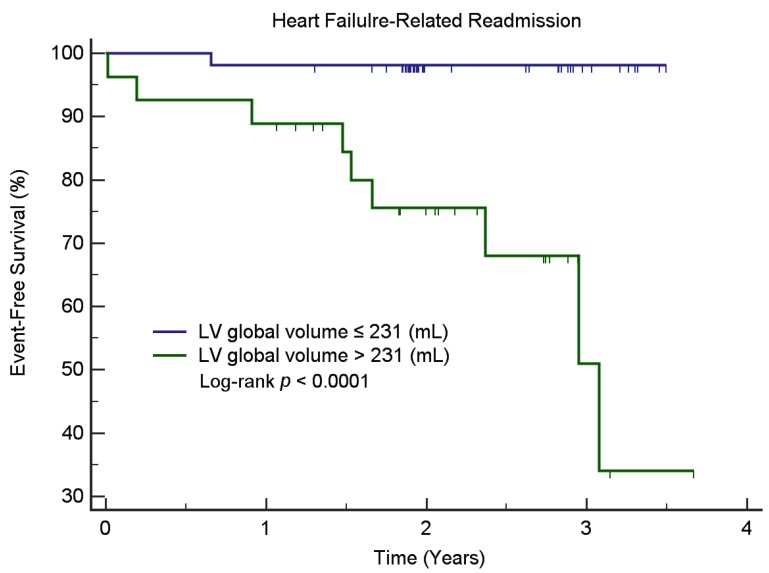
Kaplan-Meier curves for readmission-free survival in non-ischemic patients stratified according to LV (left ventricular) global volume on CMR. Note—Kaplan-Meier survival analysis showed that non-ischemic patients with low LV global volume (≤231 mL) had a lower probability for heart failure-related readmission than those with high LV global volume (>231 mL, log-rank test, *p* < 0.0001).

We found that baseline myocardial TG content—FA/UFA ratio—was significantly associated with the MACE, whilst the level of lipid resonances δ 1.6 ppm was significantly associated with HF-related readmission for those patients with ischemic heart disease. Kaplan-Meier survival analysis (Figure 4) demonstrated that ischemic patients with a low level of lipid resonance δ 1.6 ppm (≤0.99) had a lower probability for HF-related readmission than those with a high lipid resonance δ 1.6 ppm (>0.99, log-rank test, *p* < 0.0001). In non-ischemic patients, Kaplan-Meier survival analysis (Figure 5) demonstrated that patients with low LV global volume (≤231 mL) had a lower probability for HF-related readmission than those with high LV global volume (>231 mL, log-rank test, *p* < 0.0001). Myocardial TG content was not associated with MACE or HF-related readmission in non-ischemic patients. The levels of lipid resonances δ 1.6 ppm inversely correlated with the myocardial mass (r = −0.290, *p* = 0.009) and LV global volume (r = −0.282, *p* = 0.011) in non-ischemic patients. No correlations between the myocardial TG content and CMR functional parameters were found in the ischemic patient group.

## 4. Discussion

This study was designed to simultaneously assess the prognostic significance of myocardial TG content (assessed by ^1^H-MRS) and LV function parameters (measured on CMR) in the prediction of MACE and readmission in patients hospitalized for acute HF. Our main results can be summarized as follows. First, an increased LVEDVI was identified as an independent predictor of reduced MACE-free survival. Second, myocardial mass was independently associated with 3- and 12-month readmission rates. Finally, we found myocardial TG content—FA/UFA ratio—was significantly associated with MACE, whilst the level of lipid resonances δ 1.6 ppm was significantly associated with HF-related readmission for patients with ischemic heart disease. Taken together, these data indicate that assessment of LV function on CMR may improve the risk stratification of patients who have been hospitalized for acute HF. ^1^H-MRS assessment might be reserved for patients with a history of ischemic heart disease.

In patients with HF, diastolic wall strain has been reported as an independent predictor of MACE [25], and the global circumferential strain may improve the prognostic stratification [26]. However, both diastolic wall strain and global circumferential strain require expertise for post-processing from cine CMR, and were not performed in the present study. In contrast, LVEDVI (defined as the volume of blood in the LV at end load filling indexed for body surface area) is easier to integrate in the clinical routine. Mewton et al. [27] have reported that LVGFI—a CMR parameter that integrates LV structure with global function—has a strong predictive value of MACE in a multiethnic population of men and women without a history of CVD at baseline. However, in the current study, LVGFI was a significant prognostic predictor in univariate but not in multivariable analysis. It is possible that the weaker predictor value of LVGFI—as compared with LVEDVI—observed in our study could reflect compensatory modifications in LV mass and volumes aimed at preserving systolic function during HF. Because HF is a complex clinical syndrome that results from a variety of conditions preventing the LV from supporting the physiological circulation. Our study explored the possibility of linking the dysregulation of myocardial TG with the future MACE, based on evidence showing the associations of myocardial TG and CV disease [6,7,8], plus the potential of quantitative readout of MRS in differentiating various lipid species in the myocardium [10]. Using this technique, we have previously shown that patients hospitalized for acute HF are characterized by increased myocardial UFA content [13]. The predictive value of myocardial TG contents—FA/UFA ratio and levels of lipid resonances δ 1.6 ppm, were further validated in the current case-control study. Indeed, increased myocardial TG content is a prerequisite of cardiac steatosis, which may ultimately result in lipotoxicity and heart dysfunction [6,7,8,10]. In line with our results, Wei et al. [28] demonstrated that myocardial steatosis is mechanistically linked to diastolic dysfunction in women with coronary microvascular dysfunction. The early alterations of myocardial TG measured by using ^1^H-MRS might be supplementary to the diastolic dysfunction to improve the stratification for patients with ischemic heart disease.

Readmissions are frequent in patients with acute HF and served as a secondary outcome measure in the current study. A previous report demonstrated that diabetes mellitus, hyperlipidemia, CAD, length of stay at the index admission, and prescription of beta-blockers were significant predictors of readmission rates [29]. The 30-day, 3-month, and 12-month readmission rates observed in our study were 0.8%, 4.5%, 13.5%, respectively, being markedly lower than those observed in Western countries [30,31]. Interestingly, we identified myocardial mass measured on CMR as an independent predictor of readmission rates. The present study supports the concept that LV mass measured by CMR is a viable predictor of adverse cardiovascular events [27], either from the MESA (Multi-Ethnic Study of Atherosclerosis) study [32] or the Cardiovascular Health Study [33]. Further independent studies in larger sample sizes are needed to confirm this pilot observation.

Our data should be interpreted in the context of some limitations. First, the single-center of our study may bring into question its generalizability, as the study population was highly selected for those with medical conditions becoming stable and ready to discharge. The number of HF-related readmissions might be too small for meaningful multivariable analyses. However, it is noteworthy that patients were recruited regardless of the underlying cause of HF. The data should be interpreted carefully because pathophysiologically diverse endpoints were included in this study. Second, the resonance δ 1.6 ppm sometimes overlaps with the main CH_2_ resonance δ 1.3 ppm. The sum of the signal intensities δ 0.9, 1.3 and 1.6 ppm would have been more reliably extracted from an in vivo cardiac ^1^H-MRS. Nonetheless, the quantitative analysis was carried out using well-established LC Model software to enhance the generalizability of the current study. Third, longitudinal 3-T CMR and ^1^H-MRS examinations were not performed and changes in LVEDVI and/or myocardial TG content were not investigated over time. A validation cohort for our findings would help to elucidate the clinical value of such imaging biomarkers, however, it was outside of the pre-specified analysis for this study.

## 5. Conclusions

Our results indicate that LVEDVI and myocardial mass are potential imaging biomarkers that independently predict MACE and readmission, respectively, in patients discharged after hospitalization for acute HF. Myocardial TG predicts readmission in patients with a history of ischemic heart disease. Further studies are needed to determine whether LVEDVI and myocardial mass, as well as myocardial TG, may serve as therapeutic targets to improve prognoses in targeted patient populations.

## Figures and Tables

**Table 1:** Baseline characteristics of the study patients (*n* = 133).

Variable	MACE (*n* = 39)	Non-MACE (*n* = 94)	*p* Value
Clinical profile			
Male sex (%)	74.2%	57.5%	0.088
Age (years)	52.3 ± 10.0	52.7 ± 10.3	0.846
Height (m)	1.7 ± 0.1	1.7 ± 0.1	0.656
Weight (kg)	70.5 ± 13.8	69.6 ± 15.9	0.751
BMI (kg/m^2^)	25.7 ± 3.9	25.6 ± 5.3	0.953
Heart rate	73.8 ± 14.2	71.3 ± 13.2	0.350
SBP (mmHg)	125.3 ± 19.5	131.9 ± 21.1	0.087
DBP (mmHg)	73.0 ± 11.7	76.4 ± 10.2	0.116
Smoking	53.8%	42.5%	0.316
Comorbidities			
Hypertension	47.3%	45.0%	0.956
DM	23.7%	20.0%	0.813
Angina	36.6%	32.5%	0.802
MI	36.6%	32.5%	0.802
DCM	16.1%	10.0%	0.512
Myocarditis	2.2%	5.0%	0.742
CAD	35.5%	32.5%	0.894
Medications			
DM drugs	16.1%	20.0%	0.771
Anti-platelets	39.8%	37.5%	0.957
Statins	23.7%	17.5%	0.576
Thrombolytic agents	6.5%	0.0%	0.235
Antiarrhythmic drugs	14.0%	10.0%	0.729
Diuretics	39.8%	30.0%	0.381
Calcium channel blockers	10.8%	7.5%	0.794
Beta blockers	68.8%	62.5%	0.611
ACEI/ARB	68.8%	57.5%	0.289
Vasodilators	16.1%	5.0%	0.139
Iron supplements	5.4%	2.5%	0.781
Laboratory data			
AST (U/L)	31.4 ± 28.5	27.9 ± 24.3	0.556
ALT (U/L)	28.1 ± 16.5	39.8 ± 49.3	0.187
HDL (mg/dL)	43.5 ± 12.4	43.5 ± 12.1	0.988
VLDL (mg/dL)	30.3 ± 15.7	28.6 ± 13.0	0.598
LDL (mg/dL)	106.2 ± 53.2	109.7 ± 38.4	0.737
Total cholesterol/HDL	5.1 ± 5.3	4.4 ± 1.1	0.449
LDL/HDL	2.8 ± 1.2	2.7 ± 1.0	0.663
Total cholesterol (mg/dL)	189.3 ± 49.4	185.2 ± 35.7	0.672
Triglyceride (mg/dL)	165.5 ± 129.1	151.2 ± 86.2	0.564
Non-HDL (mg/dL)	145.8 ± 45.0	141.7 ± 35.4	0.662
Glucose (mg/dL)	113.2 ± 31.2	123.5 ± 51.2	0.210
HbA1c (mg/dL)	6.2 ± 1.2	6.4 ± 1.6	0.421
TIBC (ug/dL)	377.9 ± 85.8	346.7 ± 52.2	0.413
Troponin (ng/mL)	2.0 ± 8.9	0.3 ± 0.7	0.428
BNP (pg/mL)	342.9 ± 583.0	450.4 ± 569.7	0.470
Neutrophil count (%)	62.7 ± 12.6	57.5 ± 12.4	0.078
Hemoglobin (g/dL)	14.4 ± 3.5	13.5 ± 1.5	0.129
MCV (fl)	87.7 ± 6.7	86.4 ± 8.0	0.322
MCH (pg)	29.6 ± 2.7	29.0 ± 3.1	0.240
MCHC (%)	33.7 ± 1.0	41.0 ± 47.5	0.337

Note—Categorical data are expressed as numbers (%), whereas continuous variables are given as means ± standard deviations unless otherwise specified. Abbreviations: BMI, body mass index; CAD, coronary artery disease; SBP, systolic blood pressure; DBP, diastolic blood pressure; MACE, major adverse cardiac events; DM, diabetes mellitus; MI, myocardial infarction; DCM, dilated cardiomyopathy; ACEI, angiotensin-converting enzyme inhibitors; ARB, angiotensin receptor blockers; AST, aspartate aminotransferase; ALT, alanine transaminase; HDL, high-density lipoprotein; VLDL, very-low-density lipoprotein; LDL, low-density lipoprotein; TIBC, total iron-binding capacity; BNP, B-type natriuretic peptide; MCV, mean corpuscular volume; MCH, mean corpuscular hemoglobin; MCHC, mean corpuscular hemoglobin concentration.

**Table 2:** Univariate and stepwise multivariable Cox regression analysis of CMR and ^1^H-MRS factors associated with major adverse cardiovascular events.

CMR& MRS Parameters	Overall (*n* = 133)				Ischemia (*n* = 50)				Non-Ischemia (*n* = 83)			
	Univariate		Stepwise Multivariable		Univariate		Stepwise Multivariable		Univariate		Stepwise Multivariable	
Variable	HR	*p* Value	HR	*p* Value	HR	*p* Value	HR	*p* Value	HR	*p* Value	HR	*p* Value
EF (%)	0.97	<0.001			0.99	0.456			0.95	0.001		
LV EDV (mL)	1.01	<0.001			1.00	0.099			1.01	<0.001		
LV EDVI (mL/m^2^)	1.01	<0.001	1.01	<0.001 *	1.01	0.041			1.02	<0.001		
LV ESV (mL)	1.01	<0.001			1.00	0.189			1.01	<0.001		
Cardiac output (L/min)	1.08	0.463			1.25	0.067	1.32	0.044	0.95	0.768		
Myocardial mass (g)	1.01	0.001			1.01	0.144			1.01	<0.001	1.32	0.044
LV stroke volume (mL)	1.00	0.635			1.01	0.341			1.01	0.836		
LV mean cavity volume (mL)	1.01	<0.001			1.00	0.132			1.01	<0.001		
LV myocardial volume (mL)	1.01	0.001			1.01	0.144			1.01	<0.001		
LV global volume (mL)	1.00	<0.001			1.00	0.103			1.00	<0.001		
LVGFI (%)	0.97	0.001			0.99	0.562			0.95	<0.001		
FA 0.9 ppm	1.00	0.508			1.00	0.573			1.00	0.671		
FA 1.3 ppm	1.00	0.599			1.00	0.697			1.00	0.764		
FA 1.6 ppm	1.00	0.967			1.00	0.472			1.00	0.706		
UFA 2.1 ppm	1.00	0.557			1.00	0.938			1.00	0.779		
UFA 2.3 ppm	1.00	0.796			1.00	0.876			0.94	0.604		
UFA 2.8 ppm	1.00	0.796			1.00	0.753			0.18	0.772		
FA (09,13,16)	1.00	0.617			1.00	0.701			1.00	0.748		
UFA (21,23,28,53)	1.00	0.881			1.00	0.640			1.00	0.722		
TG (FA+UFA)	1.00	0.592			1.00	0.643			1.00	0.740		
FA/TG	1.68	0.219			1.89	0.292			1.60	0.605		
UFA/TG	0.60	0.219			0.53	0.292			0.62	0.605		
FA/UFA	1.00	0.089			1.01	0.023	1.01	0.022	0.98	0.426		

Note.—Abbreviations: HR, hazard ratio; CI, confidence interval; BMI, body mass index; SBP, systolic blood pressure; DBP, diastolic blood pressure; EF, ejection fraction; LV, left ventricular; EDV, end-diastolic volume; EDVI, end-diastolic volume index; ESV, end-systolic volume; FA, fatty acid; LVGFI, left ventricular global volume index; TG, triglycerides; UFA, unsaturated fatty acids. * significant after Bonferroni correction.

**Table 3:** Univariate and stepwise multivariable Cox regression analysis of CMR and ^1^H-MRS factors associated with heart failure-related readmission.

CMR& MRS Parameters	Overall (*n* = 133)				Ischemia (*n* = 50)				Non-Ischemia (*n* = 83)			
	Univariate		Stepwise Multivariable		Univariate		Stepwise Multivariable		Univariate		Stepwise Multivariable	
Variable	HR	*p* Value	HR	*p* Value	HR	*p* Value	HR	*p* Value	HR	*p* Value	HR	*p* Value
EF (%)	0.96	<0.001			0.97	0.184			0.94	0.001		
LV EDV (mL)	1.01	<0.001			1.01	0.152			1.01	<0.001		
LV EDVI (mL/m^2^)	1.02	<0.001	1.02	<0.001 *	1.02	0.106			1.02	0.001	1.01	<0.001 *
LV ESV (mL)	1.01	<0.001			1.01	0.119			1.01	<0.001		
Cardiac output (L/min)	0.85	0.348			0.92	0.781			0.81	0.326		
Myocardial mass (g)	1.01	<0.001			1.01	0.177	1.02	0.001 *	1.01	0.001		
LV stroke volume (mL)	0.99	0.447			0.99	0.699			0.99	0.408		
LV mean cavity volume (mL)	1.01	<0.001			1.01	0.130			1.01	<0.001		
LV myocardial volume (mL)	1.01	<0.001			1.01	0.177			1.01	0.001		
LV global volume (mL)	1.01	<0.001	1.01	<0.001 *	1.01	0.107			1.01	<.001	1.01	0.002 *
LVGFI (%)	0.94	<0.001			0.95	0.148			0.94	<0.001		
FA 0.9 ppm	0.70	0.523			0.86	0.708			0.00	0.555		
FA 1.3 ppm	1.00	0.746			0.96	0.832			1.00	0.769		
FA 1.6 ppm	0.68	0.635				0.006		0.003 *	0.22	0.735		
UFA 2.1 ppm	0.34	0.629			0.50	0.723			0.16	0.766		
UFA 2.3 ppm	0.65	0.718			0.82	0.737			0.24	0.770		
UFA 2.8 ppm	0.04	0.619			0.71	0.780				0.487		
FA (09,13,16)	0.99	0.589			0.96	0.0718			1.00	0.795		
UFA (21,23,28,53)	0.93	0.581			0.77	0.708			0.96	0.635		
TG (FA+UFA)	0.99	0.589			0.99	0.851			0.99	0.680		
FA/TG	2.72	0.183			56.41	0.095			1.21	0.818		
UFA/TG	0.37	0.183			0.02	0.095			0.83	0.818		
FA/UFA	1.01	0.396			1.01	0.318			1.00	0.858		

Note.—Abbreviations: HR, hazard ratio; CI, confidence interval; BMI, body mass index; SBP, systolic blood pressure; DBP, diastolic blood pressure; EF, ejection fraction; LV, left ventricular; EDV, end-diastolic volume; EDVI, end-diastolic volume index; ESV, end-systolic volume; FA, fatty acid; LVGFI, left ventricular global volume index; TG, triglycerides; UFA, unsaturated fatty acids. * significant after Bonferroni correction.

## Supplementary Materials

The following are available online at https://www.mdpi.com/2077-0383/9/1/169/s1, Table S1: CMR and 1H-MRS parameters in the study patients with and without MACE (*n* = 133).
