# Supplementary material for: Left Ventricular Function and Myocardial Triglyceride Content on 3T Cardiac MR Predict Major Cardiovascular Adverse Events and Readmission in Patients Hospitalized with Acute Heart Failure
Source: J Clin Med. 2020 Jan 8;9(1):169. doi: 10.3390/jcm9010169 (PMC7019990; doi:10.3390/jcm9010169)
Supplement: Supplementary file 1 [file jcm-09-00169-s001.pdf]

Table S1. CMR and ^{1}H-MRS parameters in the study patients with and without MACE (*n* = 133).

Variable	Entire cohort (*n* = 133)	MACE (*n* = 39)	No-MACE (*n* = 94)	*p* Value
CMR parameters				
CMR EF (%)	52.2±21.7	40.0±19.5	57.2±20.7	< 0.001
LV EDV (mL)	151.5±80.0	193.2±92.0	134.6±68.0	< 0.001
LV EDVI (mL/m^2)	85.8±43.3	109.0±50.2	76.4±36.5	< 0.001
LV ESV (mL)	84.5±74.7	126.1±86.9	67.7±62.2	< 0.001
Cardiac output (L/min)	4.8±1.8	4.8±2.3	4.8±1.6	0.958
Myocardial mass (g)	131.5±59.9	154.7±69.2	122.2±53.4	0.012
LV stroke volume (mL)	67.0±25.6	67.1±28.0	66.9±24.7	0.961
LV mean cavity volume (mL)	118.0±76.3	159.6±88.4	101.2±64.0	0.001
LV myocardial volume (mL)	125.3±57.1	147.3±65.9	116.4±50.8	0.012
LV global volume (mL)	243.3±121.7	307.0±133.4	217.5±107.1	0.001
LVGFI (%)	32.3±14.8	24.9±11.5	35.4±14.9	< 0.001
[1] H-MRS parameters				
FA (09,13,16)	202.7±1224.0	128.3±636.7	231.1±1385.6	0.674
UFA (21,23,28,53)	48.0±219.6	52.4±254.0	46.2±205.1	0.882
TG (FA+UFA)	241.6±1335.2	167.6±857.0	272.3±1492.3	0.682
FA/TG	0.6±0.4	0.6±0.4	0.6±0.4	0.293
UFA/TG	0.4±0.4	0.4±0.4	0.4±0.4	0.293
FA/UFA	14.5±31.0	20.5±42.7	12.3±25.1	0.281

Note.—Categorical data are expressed as numbers (%), whereas continuous variables are given as means ± standard deviations, unless otherwise specified. Abbreviations: EF, *ejection fraction*; LV, left ventricular; EDV, end-diastolic volume; EDVI, end-diastolic volume index; ESV, end-systolic volume; FA, fatty acid; LVGFI, left ventricular global volume index; TG, triglycerides; UFA, unsaturated fatty acids.
